# PorcineAI-Enhancer: Prediction of Pig Enhancer Sequences Using Convolutional Neural Networks

**DOI:** 10.3390/ani13182935

**Published:** 2023-09-15

**Authors:** Ji Wang, Han Zhang, Nanzhu Chen, Tong Zeng, Xiaohua Ai, Keliang Wu

**Affiliations:** 1 College of Animal Science and Technology, China Agricultural University, Beijing 100193, China; ji.wang@cau.edu.cn (J.W.); hanzhang@cau.edu.cn (H.Z.); s20223040677@cau.edu.cn (T.Z.); sy20203040845@cau.edu.cn (X.A.); 2 Institute of Animal Science, Chinese Academy of Agricultural Sciences, Beijing 100193, China; nzc1417@163.com

**Keywords:** enhancer, convolutional neural networks, sequence classification

## Abstract

**Simple Summary:**

This study develops a deep learning framework called PorcineAI-enhancer to predict enhancer sequences in pigs. Enhancers play a key role in regulating gene expression. However, identifying enhancers experimentally remains challenging. This study constructs a reliable pig enhancer dataset by integrating multiple data sources. The PorcineAI-enhancer model employs convolutional neural networks to extract features from DNA sequences and classify them into enhancers or non-enhancers. Evaluation on an independent test set shows the model has excellent performance. It also demonstrates strong predictive capability on tissue-specific enhancers from human and pig. This tool facilitates research on gene regulation mechanisms in pigs. It provides valuable resources to understand complex traits related to agriculture and biomedicine.

**Abstract:**

Understanding the mechanisms of gene expression regulation is crucial in animal breeding. Cis-regulatory DNA sequences, such as enhancers, play a key role in regulating gene expression. Identifying enhancers is challenging, despite the use of experimental techniques and computational methods. Enhancer prediction in the pig genome is particularly significant due to the costliness of high-throughput experimental techniques. The study constructed a high-quality database of pig enhancers by integrating information from multiple sources. A deep learning prediction framework called PorcineAI-enhancer was developed for the prediction of pig enhancers. This framework employs convolutional neural networks for feature extraction and classification. PorcineAI-enhancer showed excellent performance in predicting pig enhancers, validated on an independent test dataset. The model demonstrated reliable prediction capability for unknown enhancer sequences and performed remarkably well on tissue-specific enhancer sequences.The study developed a deep learning prediction framework, PorcineAI-enhancer, for predicting pig enhancers. The model demonstrated significant predictive performance and potential for tissue-specific enhancers. This research provides valuable resources for future studies on gene expression regulation in pigs.

## 1. Introduction

Understanding how complex gene expression patterns are regulated is a fundamental question in biology. At the core of this question is the genome-wide identification and characterization of cis-regulatory sequences that influence the expression of protein-coding genes and long non-coding RNA genes [1]. Cis-regulatory DNA sequences play a crucial role in the regulation of gene expression. These sequences, which can be located far away from gene promoters, have been shown to have significant effects on gene expression, sometimes resulting in up to a 100-fold increase in expression [2]. Enhancers, silencers, insulators, and tethering elements are examples of cis-regulatory sequences [3]. Among them, enhancers and their associated transcription factor proteins are particularly important in the regulation of gene expression [4]. Enhancers are genomic regions that function as major regulatory elements controlling gene expression. They play a key role in cell-type-specific gene expression programs by forming physical interactions, often over long distances, with the promoters of their target genes [5]. Multiple enhancers, located tens or hundreds of thousands of nucleotides away from their target genes, loop to their respective gene promoters and work in coordination to regulate the expression of their common target gene [5].

Enhancers can synergistically interact with functional elements such as promoters and silencers, greatly influencing the spatial and temporal expression and transcription frequency of their target genes [6]. Enhancers possess three main characteristics: firstly, enhancers exhibit specificity, meaning that each enhancer only functions in a limited number of cell types or tissues [7]. This uniqueness limits the impact of mutations on their function. Secondly, enhancers are bidirectional, meaning that enhancers can be located upstream or downstream of the promoters they activate [8]. Additionally, enhancers exhibit the feature of long-range action, where the distance between an enhancer and its activated promoter can vary [9].

These characteristics of enhancers bring about many challenges in their identification. Although high-throughput experimental techniques have achieved significant success in enhancer identification [10,11,12], the enormous number of experimental conditions resulting from the diverse activity of enhancers in different cell tissues presents a challenge [13,14,15]. It is not practical to experimentally verify the existence of all enhancers in thousands of tissues or cells, which are the two drawbacks of this method: time-consuming and expensive [16].

To overcome the limitations of high-throughput experimental techniques, computational approaches have emerged, including those based on genome comparison [17] and machine learning [18,19]. Enhancers can exist in any region of the genome, making it difficult to find a linear pattern for enhancer identification through genome comparison methods [20].

Deep learning, a hot topic in the field of machine learning, possesses powerful learning capabilities that outperform various algorithms [21,22,23]. As a result, it has been widely applied in cutting-edge disciplines such as computer vision [24,25,26] and speech recognition [27,28,29]. In the field of gene sequences, deep learning has been proven to be a highly effective method for enhancer prediction [16,30,31,32,33,34,35]. Unlike traditional machine learning methods, deep learning constructs multi-layer neural networks to learn feature representations, enabling the automatic learning of higher-level abstract features [36] and better handling of nonlinear data [37]. Therefore, deep learning methods can leverage more information for enhancer prediction while reducing reliance on feature engineering. In recent years, deep learning methods have achieved significant success in predicting human enhancers [38,39,40] and have gradually become one of the methods for enhancer prediction.

In animal husbandry, to maintain food and agricultural production while minimizing negative environmental impacts, understanding the molecular mechanisms underlying economically important complex traits in farm animals is crucial for achieving biology-driven breeding biotechnologies. The domestic pig (Sus scrofa), a cornerstone of economic food security and international trade for many countries, holds significant positions in both animal husbandry and the biomedical field. Identifying enhancer regions in pigs bears great importance for advancing livestock farming and biomedical research. Enhancers play a pivotal role in gene regulation, and linking them with genes, SNPs, SVs, or other regions of interest holds the promise of providing valuable insights into the regulation of complex production traits and adaptive characteristics. It’s worth emphasizing that pigs are not only of interest due to their production traits but also due to their physiological similarities with humans. They are widely used as large animal models for preclinical research [41,42] and as xenotransplantation donors [43,44]. These features make pigs vital resources in the field of research, rendering a deeper understanding of the regulatory mechanisms of the pig genome immensely valuable for driving scientific research and applications.

Some studies [45,46,47,48] have utilized two enhancer-associated histone modifications, H3K27ac and H3K4me1 [7,49,50,51]. However, these studies come with high implementation costs, are limited by tissue/cell types and genetic backgrounds, and still require substantial effort and funding to determine whether these regions indeed possess actual enhancer functionality, as demonstrated through regulatory assays in transgenic mice [52].

While there have been cost-saving efforts in using deep learning to predict enhancer sequences in humans and mice [53,54,55], the progress in predicting enhancer sequences in livestock remains relatively limited due to the lack of a publicly accessible and reliable enhancer database for livestock species. As a result, deep learning models have not been widely applied to predict enhancer sequences in livestock, particularly in poultry and other livestock species.

In this study, we took the first step in addressing this issue by utilizing a publicly available pig enhancer database to construct a trustworthy dataset of enhancer and non-enhancer sequences. Subsequently, we employed this dataset to train a deep learning framework, named PorcineAI-enhancer, for enhancer prediction in pig genomic sequences. The main idea behind this model is to combine one-hot encoding and k-mer encoding to represent sequence data and then use CNN to extract features and perform classification, thus determining whether a sequence belongs to an enhancer region. Experimental results on an independent test dataset demonstrate the excellent performance of this method.

We have made our PorcineAI-enhancer code and data freely available on GitHub repository: https://github.com/castwj/PorcineAI-enhancer (accessed on 10 August 2023), facilitating accessibility and encouraging further research and collaboration in this field.

## 2. Materials and Methods

### 2.1. Data Preparation

Although several scientists have conducted ChIP-seq experiments to explore enhancer elements in pigs [47,56,57,58], most of these experiments lack collaborative support from other high-throughput data and functional validation in the laboratory. This phenomenon has resulted in a limited number of reliable enhancers in pigs, with varying quality.

To construct a deep learning model, there is an urgent need to create a high-quality and highly reliable enhancer database for pigs. In this study, we collected relevant enhancer sequence information from three different sources. Firstly, MacPhillamy et al. [59] utilized transfer learning methods and high-quality enhancer data from VISTA [52] and publicly available human and mouse ChIP-seq data to study enhancer functionality in three non-model mammalian species (cattle [45,60,61,62], pigs [45,47], and dogs [62]). By combining this data with species-specific ChIP-seq data, they obtained a high-confidence enhancer list. Secondly, the Functional Annotation of Animal Genomes (FAANG) project [63] is an international collaborative initiative aimed at systematically annotating animal genomes. The project employs a variety of high-throughput techniques and bioinformatics methods to comprehensively annotate the genomes of various animal species, revealing their functions and regulatory mechanisms. Recently, Pan et al. [64] integrated 223 epigenomic and transcriptomic datasets to create a comprehensive catalog of regulatory elements in pigs (Sus scrofa). We extracted enhancer information from this catalog for all tissues and merged it. Additionally, the EnhancerAtlas 2.0 database [65] is a multi-species public database that contains 13,494,603 enhancers from 16,055 datasets.

The aforementioned three datasets collectively constitute the enhancer data sources used in this study. It’s worth noting that the EnhancerAtlas 2.0 database [65] uses the Sscrofa10.2 reference genome, while the other two datasets use Sscrofa11.1 [66]. Since the conversion of BED files between different reference genomes can potentially lead to the loss of some sequences, we opted to use pig iPSC and heart enhancer information from the EnhancerAtlas 2.0 database [65] as our test data. This choice aims to assess the PorcineAI-enhancer model’s ability to recognize tissue-specific enhancers. Additionally, the EnhancerAtlas provides human iPSC and heart enhancer information (using hg19 as the reference genome). Considering the relatively close genetic relationship between humans and pigs, we can compare cross-species tissue-specific enhancer prediction capabilities to explore the model’s reliability.

### 2.2. High Confidence Sequence Acquisition

To construct an effective and robust model, we followed strict criteria to establish the dataset. To obtain high-quality and reliable enhancer sequences, we combined enhancer sequences obtained from MacPhillamy et al. [59] and Pan et al. [64]. We processed the BED files from these two datasets to obtain the overlapping fragments, which served as the initial enhancer sequences. We retained only sequences with a length more than 200 bp. Then, based on the length requirements of model, we divided the sequences into fixed-length (200 bp) fragments. Sequences shorter than 200 bp were discarded. We used Bedtools [67] to obtain the sequences, resulting in a total of 7633 enhancer sequences.

**Figure 1:**
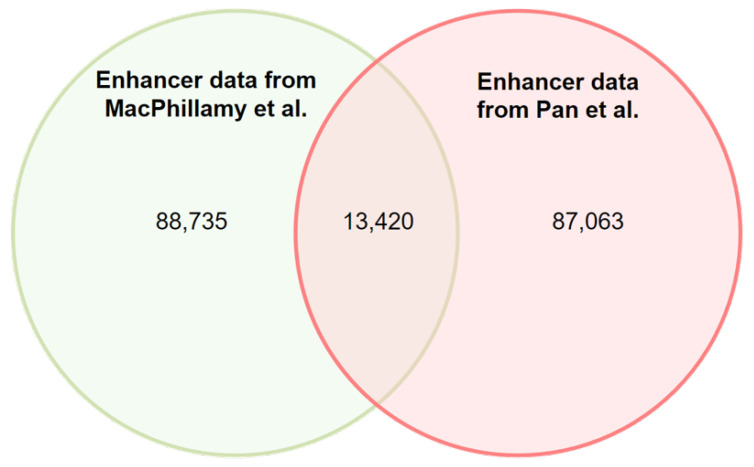
Enhancer Source Venn Diagram. Each circle representing a specific source, the overlapping regions indicate the common enhancers shared between the sources, while the non-overlapping regions represent the unique enhancers specific to each source. MacPhillamy et al. [59] and Pan et al. [64].

To provide an intuitive representation of the enhancer dataset used by the model within the context of the original datasets, we employed an enhancer source Venn diagram, Figure 1. This diagram effectively illustrates the overlapping and non-overlapping portions of enhancers from different sources.

Regarding the non-enhancer sequences, previous studies often randomly extracted genomic fragments as negative samples [30], ensuring that their length distribution and quantity were the same as the enhancer sequences. However, considering the potential misclassification of some tissue-specific enhancers as non-enhancer sequences due to experimental design limitations, to ensure the reliability and practicality of the non-enhancer sequences, we initially utilized the gene annotation file of Sus11.1 [66] to extract fundamental genomic information. This encompassed gene annotations (such as protein-coding genes and long non-coding RNAs) and promoter regions (defined as 2 kb regions centered around the transcription start site of protein-coding genes). These sequences were intentionally selected as they represent regions that are unlikely to be enhancers.

We then combined these sequences with the enhancer sequence regions covered by the databases employed in our study. Using Bedtools [67], we filtered out the remaining genomic regions. Lastly, we randomly selected 7633 segments from these filtered regions to constitute the non-enhancer dataset used for training.

To reduce sequence similarity, we employed the Cd-hit [68,69,70] tool to remove redundant sequences with a similarity exceeding 80%. Finally, we used the resulting non-redundant sequences as the samples for the reference dataset.

### 2.3. Sequence Coding Method

In many deep learning algorithms used for processing biological sequences, natural language processing techniques are commonly employed to extract features from raw DNA sequences [71,72,73]. In our CNN model, we utilized a method called One-hot Encoding and k-mer descriptors to encode each input sequence. Each enhancer sequence in this study consists of four bases, adenine (A), guanine (G), cytosine (C), and thymine (T), with a length of 200 bp.

In the One-hot Encoding of genetic sequences, we represent each base as a one-hot vector of length four, where only one element is 1, and the rest are 0. For example, adenine (A) is represented as [1, 0, 0, 0], cytosine (C) is represented as [0, 1, 0, 0], and so on. Thus, the genetic sequence can be represented as a concatenation of a series of one-hot vectors, where each one-hot vector represents a base.

K-mer encoding is a method for converting protein or DNA sequences into vector representations. It treats every consecutive k characters (or letters) in the sequence as a unit and represents each unit as a numeric vector. When the step size is 1, a DNA sequence of length l can be divided into (l − k + 1) k-mers. For example, when k = 2, the sequence ‘ACGTCGACG’ will be divided into seven 2-mers: “AC”, “CG”, “GT”, “TC”, “GA”, “AC”, “CG”. This representation makes the sequence easier to compute and understand. We treat the entire DNA sequence as a sentence and the k-mer fragments as words. These vectors can be used for various bioinformatics tasks such as classification, clustering, sequence alignment, and pattern recognition.

One drawback of k-mer encoding is that it may lose some contextual information of the sequence since it divides the sequence into independent k-mer units. Additionally, k-mer encoding can be influenced by the sequence length and the chosen value of k, requiring optimization based on specific circumstances. To mitigate the impact of k-mer encoding on the results, in this study, we set the values of k to 1, 2, and 3 respectively, to strike a better balance between contextual information and computational efficiency.

To combine the One-hot and k-mer representations and form the inputs to our model, we concatenated them together, resulting in a comprehensive feature vector that captures both the nucleotide composition and sequential patterns present in the DNA sequence. This hybrid approach enables us to leverage the fine-grained information captured by the one-hot encoding and the higher-order patterns captured by the k-mer encoding simultaneously.

Specifically, the shape of the concatenated feature vector is (4 + 1 + 2 + 1) × SAMPLE LENGTH, where the first four rows correspond to the one-hot encoding of the sequence, the fifth row corresponds to the 1-mer features, the sixth and seventh rows correspond to the 2-mer features considering both left and right directions, and the last row corresponds to the 3-mer features. This comprehensive feature representation effectively captures the individual nucleotide composition and higher-level sequence patterns present in the enhancer sequence. SAMPLE LENGTH represents the chosen length of the sequence.

Finally, we convert this concatenated feature vector into a PyTorch tensor and use it as input, along with the corresponding labels, to the neural network model. This enables the model to learn from the combined information of one-hot and k-mer representations and make accurate predictions.

### 2.4. Sequence Analysis

Sequence analysis is a computational approach used to analyze biological sequences, such as DNA, RNA, and protein sequences. It helps researchers understand the patterns and structures of biological sequences and enables analysis and comparison of these sequences to reveal information about their functions, structures, and evolution.

SeqLogo [74] is a commonly used sequence analysis tool for visualizing conservation and variation information in DNA, RNA, or protein sequences. SeqLogo graphs typically represent the information entropy of each base or amino acid at each position in the sequence using the height of the corresponding letter. Higher information entropy indicates less conservation at that position.

In a SeqLogo graph, the height at each position reflects the distribution of different bases or amino acids at that position. If a specific base or amino acid is highly prevalent (high frequency) at a particular position, the height at that position will be higher, indicating higher conservation. Conversely, if there are multiple different bases or amino acids at a certain position, the height will be lower, indicating higher variation.

By visualizing conservation and variation through SeqLogo graphs, researchers can quickly gain insights into the conservation and variation within a sequence, aiding in the analysis and interpretation of its function and structure. SeqLogo graphs are commonly used to identify conserved motifs, functional sites, and important sequence features.

### 2.5. CNN Model Architecture

The PorcineAI-enhancer model we propose is a convolutional neural network designed for identifying pig genomic enhancer and non-enhancer sequences. The model consists of two convolutional blocks, each comprising three convolutional layers followed by a batch normalization layer, with a max-pooling layer after each convolutional block. The first convolutional block has 32 output channels for its convolutional layers, with a kernel size of 4 × 4 and a padding of 1. The second convolutional block is similar to the first one, but with an increased output channel size of 64.

After the convolutional blocks, the model flattens the output and processes it through a fully connected layer with a ReLU activation function and a size of 256. Finally, the output passes through a sigmoid activation function and a linear layer to generate scalar output.

The model utilizes binary cross-entropy loss as the training criterion. The forward method of the model takes an input tensor and outputs a tensor of the same size, representing the predicted output for each input sample. The model is trained through backpropagation and optimization algorithms to minimize the loss function and improve the accuracy of predictions. Table 1 below shows the variations in model parameters across the layers of the CNN model.

These parameter variations provide insights into the number of parameters in the model and the shape changes between layers. This aids in understanding the complexity of the model and the distribution of parameters, as well as the changes that may occur during the training and optimization processes.

### 2.6. K-Fold Cross-Validation

K-fold cross-validation is a commonly used model evaluation method [75]. It involves splitting the dataset into k non-overlapping subsets or folds, and then iteratively using each fold as the validation set and the remaining k-1 folds as the training set to train the model. The evaluation results from each iteration are then aggregated to obtain the average performance of the model.

K-fold cross-validation can effectively reduce overfitting by utilizing more data for model training and providing a comprehensive evaluation of the model’s performance. Additionally, it helps in selecting the best model hyperparameters, such as regularization parameters and learning rates, to improve the model’s generalization ability.

**Figure 2:**
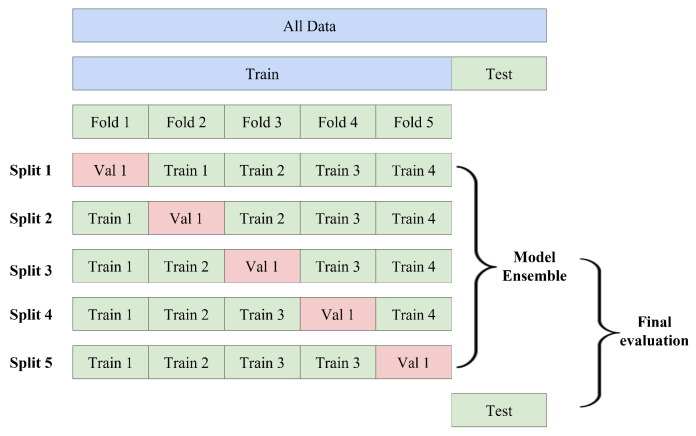
Training and Validation Process for PorcineAI-Enhancer Model using Stratified Sampling and Ensemble Learning. This figure illustrates the training and validation process for the PorcineAI-Enhancer model. The training set is randomly divided into five folds or partitions using stratified sampling, allowing for a balanced representation of the data in each fold. Each fold is then used as the validation set in turn, while the remaining four folds are used as the training set for training the Convolutional Neural Network (CNN) model. The five trained CNN models are combined to form an ensemble model, which is used to test the samples in an independent test set. This entire process, including data partitioning, model training, and model testing, is repeated five times to observe the variation in model performance across the five experiments. The use of stratified sampling and ensemble learning helps to improve the accuracy and robustness of the PorcineAI-Enhancer model.

In the training of the PorcineAI-enhancer model, we first randomly divide the training set into five folds or partitions using stratified sampling, as illustrated in Figure 2. Each fold is used as the validation set in turn, while the remaining four folds are used as the training set for training the CNN model. Then, the five trained CNN models are combined to form an ensemble model. Next, the ensemble model is used to test the samples in an independent test set. This entire process, including data partitioning, model training, and model testing, is repeated five times to observe the variation in model performance across the five experiments.

By employing k-fold cross-validation, we can comprehensively evaluate the performance of the PorcineAI-enhancer model and observe how it performs with different combinations of training and validation sets. This approach helps obtain more reliable performance evaluation results and provides guidance for further improvements to the model.

## 3. Results

### 3.1. Sequence Analysis

In the SeqLogo plot, the vertical axis can be scaled using frequency or bits. When the frequency is used as the vertical axis, the SeqLogo plot displays the frequency of occurrence for each type of base or amino acid at each position. The higher the frequency, the taller the letter, indicating a more conserved base or amino acid at that position. Conversely, the lower the frequency, the lower the letter, indicating a more variable base or amino acid at that position. When bits are used as the vertical axis, the SeqLogo plot represents the information entropy of bases or amino acids at each position. The higher the information entropy, the taller the letter, indicating a less conserved base or amino acid at that position.

**Figure 3:**
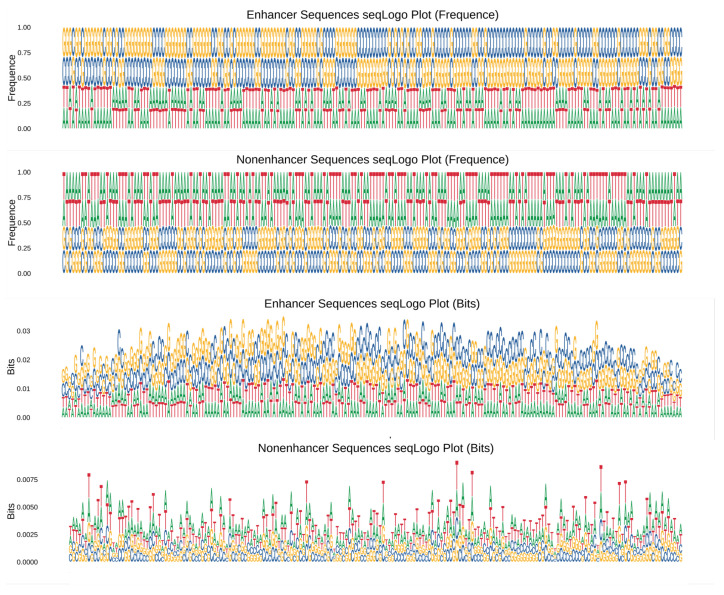
Differences in Information Entropy of Enhancer and Non-Enhancer Sequences Revealed by SeqLogo Analysis. In this figure, we show the results of SeqLogo analysis, which is a graphical representation of the conservation and variation of nucleotide or amino acid sequences. The vertical axis of the SeqLogo plot can be scaled using frequency or bits. Our analysis reveals that enhancer sequences and non-enhancer sequences exhibit significant differences in their information entropy when bits are used as the vertical axis. This indicates that enhancer sequences and non-enhancer sequences possess distinct characteristics in terms of sequence conservation and variation, which may be associated with their different roles in gene expression regulation. These findings provide further insights into the functional differences between enhancer and non-enhancer sequences and may have implications for understanding the mechanisms of gene expression regulation.

Our results are presented in Figure 3. When the frequency is used as the vertical axis, the distribution of enhancer sequences and non-enhancer sequences is nearly identical. However, when bits are used as the vertical axis, they exhibit noticeable differences in their distribution.

These findings suggest that there are significant differences in the information entropy between enhancer and non-enhancer sequences. This indicates that enhancer sequences and non-enhancer sequences possess distinct characteristics in terms of sequence conservation and variation. These characteristics may be associated with their different roles in gene expression regulation.

### 3.2. PorcineAI-Enhancer Model Training

**Figure 4:**
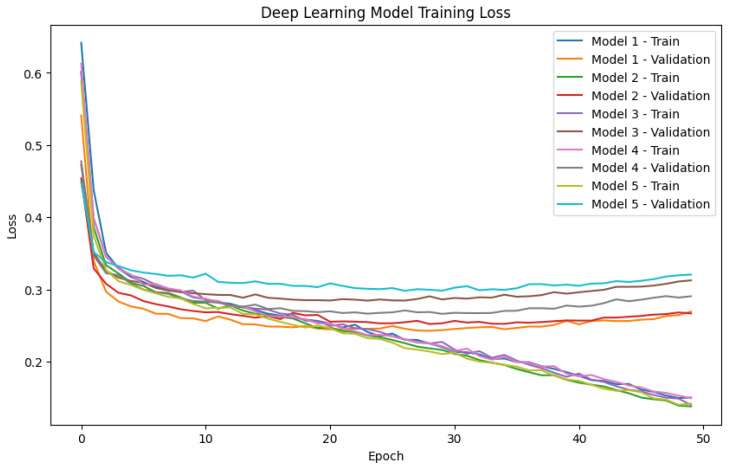
PorcineAI-enhancer model training loss curves. The horizontal axis represents the number of training epochs, and the vertical axis represents the model’s loss value. The loss value is a metric that measures the difference between the model’s predictions and the actual labels. Our goal is to minimize the loss value through training. We observe two different loss curves. One is the loss curve on the training set, which indicates the model’s fit to the training data. The other curve is the loss curve on the validation set, which represents the model’s performance on unseen data. We use the validation set to evaluate the model’s generalization ability in real-world scenarios. Typically, we select the epoch corresponding to the minimum validation set loss as the optimal model’s parameters.

We conducted model training for the PorcineAI-enhancer model. As depicted in the Figure 4, it provides a more intuitive overview of the training process for the PorcineAI-enhancer model. As illustrated in Figure 1, Model 1 refers to the training configuration where data from Fold 2–5 is employed as the training dataset, and Fold 1 serves as the validation dataset. The model is built using the parameters that exhibit the best performance on the validation set. Similarly, Model 2–5 follow this pattern, each involving a specific fold for validation while the remaining folds are utilized for training. A total of 50 epochs, where each epoch represents a complete iteration through the dataset, were carried out for training. Throughout the training process, a learning rate of 1 × 10${}^{-5}$ was utilized—an essential hyperparameter controlling the step size for model parameter updates. To optimize the model’s training, the Adam optimizer was chosen. Adam is a commonly used adaptive learning rate optimization algorithm that dynamically adjusts the learning rate based on estimates of the first and second moments of the gradients.

From the figure, it is observable that after around 20 epochs, the model’s performance on the validation set had already reached its peak. This indicates that while the model might potentially achieve better scores on the training set, further training is detrimental to performance improvement. This phenomenon suggests the occurrence of overfitting, where the model overly adapts to the training data and subsequently performs poorly on new data. To counteract overfitting, ensuring the model’s generalization ability, we opted to utilize the parameters from the epoch at which each model performed best on the validation set as the parameters for the Ensemble model. This approach enables us to attain better predictive performance on previously unseen data, enhancing model stability and reliability.

### 3.3. Performance of the PorcineAI-Enhancer Model

Through 5-fold cross-validation on the training set, we obtained 5 validated CNN models. These models were then evaluated on independent test sets, and the evaluation parameters are presented in Table 2.

From Table 2, it can be observed that the accuracy of the models ranges from 0.905 to 0.911, with a very small standard deviation, indicating their ability to accurately classify samples. As for the AUC metric, all values exceed 0.939, with the highest AUC value being 0.946, demonstrating the models’ high capability in discriminating between positive and negative samples. The higher AUC values suggest effective classification of positive and negative samples and demonstrate strong predictive performance.

**Figure 5:**
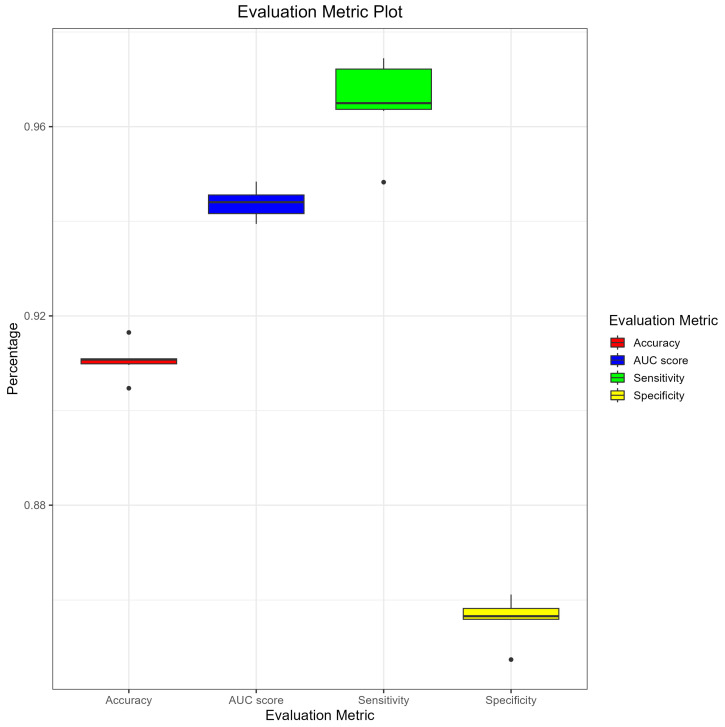
Robust Performance of Deep Learning Models in Predicting Enhancer Sequences. We present the evaluation metrics of five deep learning models in predicting enhancer sequences. The models demonstrate high accuracy and AUC values, indicating their capability in discriminating between positive and negative samples. The evaluation metrics exhibit consistent distribution, with specificity being the lowest, which may be attributed to the presence of false negatives in the non-enhancer sequences. Nevertheless, all models possess sufficient capability to predict whether a sequence is an enhancer, demonstrating the reliability of our construction of the original training data. These findings support the effectiveness and feasibility of the proposed method and highlight the robustness of the features and patterns learned by the deep learning models during the training process. The robust performance of the models suggests their potential applications in predicting enhancer sequences and advancing our understanding of gene expression regulation.

By referring to Figure 5, we can observe that the evaluation metrics of the five models exhibit consistent distribution, with specificity being the lowest. Considering that our acquisition of non-enhancer sequences did not undergo experimental verification but rather aimed to remove known functional sequences, the lower specificity may be attributed to the presence of false negatives in the non-enhancer sequences. Nevertheless, the evaluation parameters of all models indicate that each model possesses sufficient capability to predict whether a sequence is an enhancer, underscoring the reliability of our construction of the original training data.

These excellent evaluation metrics further substantiate the effectiveness and feasibility of the proposed method. The models achieve satisfactory results across multiple indicators, highlighting the robustness of the features and patterns learned by the deep learning models during the training process.

### 3.4. Comparison with Ensemble Model

Given the excellent sequence prediction capabilities exhibited by each individual model, but with some variations, we decided to further improve the predictive performance by using model ensembles. The advantage of ensemble models lies in their ability to leverage the strengths of multiple models, resulting in higher accuracy and stronger discrimination. By combining the effects of the ensemble models, we can obtain more reliable and stable prediction results.

Therefore, we constructed an ensemble model using the predictions from each individual model, and its model evaluation parameters are presented in Table 2. It is evident that the ensemble model outperforms the individual models in terms of accuracy score and AUC metrics. The ensemble model also demonstrates advantages in terms of sensitivity and specificity. The sensitivity of the ensemble model is the same as the best individual model, both achieving a value of 0.9745. This indicates that the model is highly sensitive in detecting true positive samples and avoids misclassifying them as negative samples. This is crucial in many real-world scenarios where the focus is on true positive cases. From the perspective of these evaluation metrics, the ensemble model exhibits significant advantages over the individual models, providing more reliable and accurate predictive performance.

**Figure 6:**
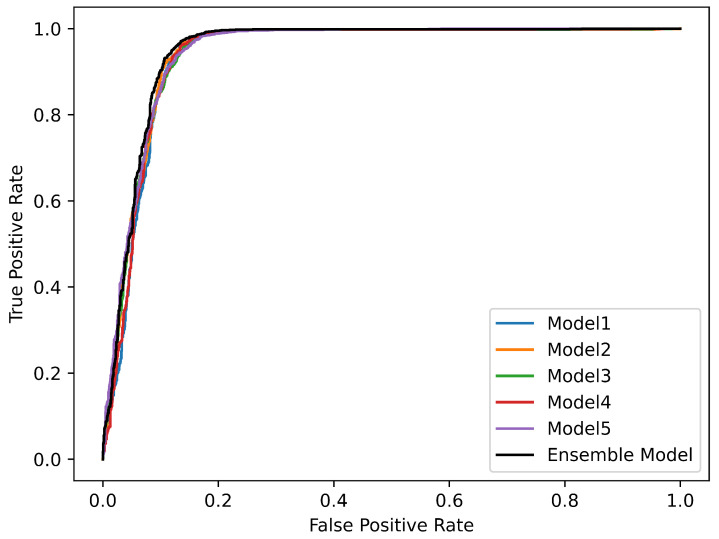
AUC Curves of Different Models. AUC score (Model 1 = 0.939438503, Model 2 = 0.944208139, Model 3 = 0.94386183, Model 3 = 0.940875633, Model 3 = 0.94601431, Ensemble Model = 0.948383796). A higher AUC score signifies that the model performs better across the entire range of decision thresholds, demonstrating its strong discriminative capability and overall effectiveness in distinguishing between positive and negative samples.

The Figure 6 below presents the AUC curves plotted for each model on the test set. The AUC curve is a common tool for evaluating the performance of classification models. It illustrates the relationship between the true positive rate (Sensitivity) and the false positive rate (1-Specificity) at various thresholds. A value closer to 1 indicates superior performance of the model in classification tasks. Upon examining this graph, it is evident that the AUC curve of the Ensemble model slightly surpasses those of the other models. This indicates that the Ensemble model maintains a better balance between the true positive rate and the false positive rate at various thresholds.

These results further validate the effectiveness and feasibility of our proposed method. The ensemble model achieves satisfactory results across multiple metrics, showcasing the robustness of the features and patterns learned by the deep learning model during the training process. This also supports our research hypothesis and provides strong evidence for a deeper understanding of gene expression regulation.

### 3.5. Comparison with Existing State-of-the-Art Methods

While the current state of enhancer prediction in pigs lacks documented research, a wealth of related studies exists in the human domain. To underscore the advancements of the PorcineAI-enhancer model, we chose to compare it with other renowned models in the realm of human enhancer prediction. Evaluation of the model’s performance was conducted by analyzing several key metrics, as depicted in the Table 3 below.

As anticipated, the model without fine-tuning exhibited noticeably lower accuracy in predicting enhancers compared to all models based on human enhancer data. While the accuracy in predicting non-enhancers was relatively higher, this is primarily due to the PorcineAI-enhancer model’s inclination to label all human sequences as non-enhancers, thereby inflating its performance in non-enhancer prediction.

Upon training the model with human enhancer data, significant improvements across all metrics were observed for the PorcineAI-enhancer model, particularly in terms of AUC and MCC. Furthermore, when compared to the iEnhancer-EBLSTM method from 2021, our study’s model slightly outperformed in terms of SN and MCC, but exhibited slightly lower performance in ACC, AUC, and SP. The comparative outcomes highlight that the PorcineAI-enhancer model excels over previous methods in many aspects. This superiority can be attributed to the inherent strengths of deep learning models, which are capable of more accurately capturing features and achieving higher efficiency in learning processes. The resultant model has more precise parameters, thereby achieving superior performance outcomes. This further substantiates the effectiveness and potential of our proposed approach in enhancer prediction.

### 3.6. Model Performance on Tissue-Specific Enhancers

The generalization performance of deep learning models holds immense importance in practical applications. Due to the strong nonlinear fitting capability of deep learning models, issues such as training overfitting [79] and insufficient training data [80] can lead to a situation where the model performs well on the training data but poorly on new data. Overfitting causes the model to only capture the features of the training dataset without abstracting more general features, resulting in a loss of predictive ability in real-world scenarios [81]. Consequently, selecting an appropriate training set and effective model training are critical issues in the field of deep learning.

In this study, we meticulously selected enhancer sequences spanning different species and tissues as our training set to assess the model’s generalization ability. These tissue-specific enhancer datasets originated from the EnhancerAtlas 2.0 database [65], encompassing enhancer sequences from human and pig iPSCs as well as heart cells. We employed the trained ensemble model to predict these sequences and compiled the prediction results in Table 4.

Analyzing the data in Table 4, it’s evident that the model maintains remarkable generalization performance in heart tissue, achieving prediction accuracies of 0.8240 and 0.7031 for pig and human heart enhancer sequences, respectively. These outcomes explicitly demonstrate the model’s generalization capacity, performing well even on test data, indicating that the model isn’t merely overfitting to the training data. These results not only enhance our confidence in applying the model to a broader range of pig cell tissues but also establish a solid foundation for cross-species enhancer prediction.

However, it’s noteworthy that the accuracy of the model in predicting pig and human iPSC enhancer sequences is relatively lower, at 0.2606 and 0.3146, respectively. Importantly, iPSCs are undifferentiated cell types, and their gene regulatory mechanisms might differ significantly from those of mature tissue cells. Therefore, the model’s suboptimal performance on such cells doesn’t necessarily reflect a weak generalization capability of the model. This further underscores the limitations of the model’s applicability and provides insights for future improvements.

## 4. Discussion

We proposed the PorcineAI-enhancer framework, which leverages deep learning techniques and addresses the challenge of limited high-quality datasets, providing a valuable tool for predicting enhancers in pigs. The development of this framework, along with the construction of a high-quality enhancer database specifically tailored for pigs, represents a significant contribution to the field of enhancer prediction. However, the framework still has some limitations and potential areas for improvement.

Through performance evaluation on an independent test dataset, the PorcineAI-enhancer framework demonstrates excellent performance in enhancer prediction, showcasing its potential in predicting pig enhancers. These findings align with previous studies in human enhancer research [82,83,84,85], which indicate the effectiveness of deep learning in predicting enhancers across various species, including humans. Therefore, similar to model organisms such as humans, applying deep learning approaches to identify gene regulatory elements in livestock genomes could become a new paradigm in livestock breeding [86,87].

However, the dataset used in this study was constructed by integrating enhancer sequence information from various sources [59,64,65], including transfer learning methods, publicly available ChIP-seq data, and comprehensive regulatory catalogs. While we made efforts to ensure the reliability and quality of the data, potential biases and inconsistencies may still exist, particularly in the case of non-enhancer sequences where the random sampling approach we employed may have limitations. Hence, future research should consider incorporating more high-quality and well-validated enhancer datasets and non-enhancer datasets to further enhance the accuracy and generalizability of the framework.

Furthermore, we must acknowledge that although the PorcineAI-enhancer framework performs well in practical applications, this study still has its limitations. Firstly, due to the diversity of pig breeds [56], tissue specificity [57,88], and developmental stages in reality, further validation and verification of the enhancer prediction capability need to be conducted under controlled conditions to ensure the reliability of the predictions. This represents the next step for model improvement, namely fine-tuning by incorporating Chip-seq-detected enhancer sequences from different breeds, tissues, and cells, expanding its applicability to more refined application scenarios. By utilizing enhancer sequence data from different breeds, we can better understand the conservation and diversity of enhancer sequences across different species.

Additionally, our deep learning model has been widely used and performed well in previous studies. However, other deep learning model frameworks, such as attention mechanisms [89], can be employed to capture longer, more complex, and higher-level sequence features. From this perspective, further improving the PorcineAI-enhancer framework to enhance its performance represents a developmental direction for increasing the predictive capabilities of the model.

## 5. Conclusions

In conclusion, this study presents the development and evaluation of the PorcineAI-enhancer framework, a deep learning-based approach for enhancer prediction in pigs. The framework demonstrates excellent performance in identifying enhancer sequences and addresses the lack of high-quality datasets specific to pigs. The findings highlight the potential of deep learning techniques in enhancer prediction and contribute to the growing body of evidence supporting their effectiveness across species. The framework provides a valuable tool for researchers studying pig gene regulation and expression patterns, facilitating advancements in understanding the molecular mechanisms underlying pig traits and diseases. Despite the limitations and the need for further validation and improvement, the PorcineAI-enhancer framework represents a significant advancement in the field and sets the stage for future studies aiming to unravel the regulatory landscape of pigs and other species.

## Figures and Tables

**Table 1:** Variations in CNN Model Parameters Across Layers.

Layer (Type)	Output Shape	Param
Conv1d-1	[−1, 32, 200]	800
BatchNorm1d-2	[−1, 32, 200]	64
Conv1d-3	[−1, 32, 200]	3104
BatchNorm1d-4	[−1, 32, 200]	64
Conv1d-5	[−1, 32, 200]	3104
BatchNorm1d-6	[−1, 32, 200]	64
MaxPool1d-7	[−1, 32, 50]	0
Conv1d-8	[−1, 64, 50]	6208
BatchNorm1d-9	[−1, 64, 50]	128
Conv1d-10	[−1, 64, 50]	12,352
BatchNorm1d-11	[−1, 64, 50]	128
Conv1d-12	[−1, 64, 50]	12,352
BatchNorm1d-13	[−1, 64, 50]	128
MaxPool1d-14	[−1, 64, 12]	0
Linear-15	[−1, 256]	196,864
Linear-16	[−1, 1]	257

**Table 2:** Performance Evaluation of CNN Models for Enhancer Prediction.

Model	Accuracy Score	AUC Score	Sensitivity	Specificity
Model 1 (Parts 2, 3, 4, 5: Part 1)	0.909626719	0.939438503	0.963326785	0.855926654
Model 2 (Parts 1, 3, 4, 5: Part 2)	0.910936477	0.944208139	0.974459725	0.847413229
Model 3 (Parts 1, 2, 4, 5: Part 3)	0.910609037	0.94386183	0.965291421	0.855926654
Model 4 (Parts 1, 2, 3, 5: Part 4)	0.910936477	0.940875633	0.964636542	0.857236411
Model 5 (Parts 1, 2, 3, 4: Part 5)	0.904715128	0.94601431	0.948264571	0.861165684
Ensemble Model	0.916502947	0.948383796	0.974459725	0.858546169

**Table 3:** Result of comparison with existing state-of-the-art methods.

Method	ACC	AUC	SN	SP	Source
iEnhancer-2L	0.730	0.806	0.710	0.750	Liu et al., 2016 [30]
EnhancerPred	0.740	0.801	0.735	0.745	Jia and He, 2016 [76]
iEnhancer-EL	0.748	0.817	0.710	0.785	Liu et al., 2018 [77]
iEnhancer-EBLSTM	0.772	0.835	0.755	0.795	Niu et al., 2021 [78]
PorcineAI-enhancer	0.652	0.811	0.335	0.969	This study
PorcineAI-enhancer (human enhancer data)	0.769	0.832	0.785	0.752	This study

**Table 4:** Performance of the Ensemble Model on Tissue-Specific Enhancer Datasets.

Tissue	Pig	Human
Heart	0.8240	0.7031
iPSC	0.2606	0.3146

## Data Availability

The source code and data can be freely accessed from the GitHub repository: https://github.com/castwj/PorcineAI-enhancer (accessed on 10 August 2023).
